# Reaffirming the importance of nomenclature stability for *Candida auris* and its associated disease of candidiasis

**DOI:** 10.1128/jcm.01550-25

**Published:** 2025-12-23

**Authors:** Sean X. Zhang, Sybren de Hoog, David W. Denning, Sarah A. Ahmed, Ana Alastruey-Izquierdo, Maiken Cavling Arendrup, Andrew Borman, Sharon Chen, Anuradha Chowdhary, Robert C. Colgrove, Oliver A. Cornely, Philippe J. Dufresne, Laura Filkins, Jean-Pierre Gangneux, Josepa Gené, Andreas H. Groll, Jaques Guillot, Gerhard Haase, Catriona Halliday, David L. Hawksworth, Roderick Hay, Martin Hoenigl, Vit Hubka, Tomasz Jagielski, Hazal Kandemir, Sarah E. Kidd, Julianne V. Kus, June Kwon-Chung, Shawn R. Lockhart, Jacques F. Meis, Leonel Mendoza, Wieland Meyer, M. Hong Nguyen, Yinggai Song, Tania C. Sorrell, J. Benjamin Stielow, Raquel Vilela, Roxana G. Vitale, Nancy L. Wengenack, P. Lewis White, Luis Ostroski-Zeichner, Thomas J. Walsh, members of the ISHAM/ECMM/FDLC Working Group Nomenclature of Clinical Fungi

**Affiliations:** 1 Johns Hopkins Hospital & Johns Hopkins University School of Medicine 1500 , Baltimore, Maryland, USA; 2 Radboudumc-CWZ Centre of Expertise for Mycology 443325 , Nijmegen, the Netherlands; 3 Foundation Atlas of Clinical Fungi, Hilversum, the Netherlands; 4 Manchester Fungal Infection Group, Academic Health Science Centre, University of Manchester 5292, Manchester, United Kingdom; 5 Department of Microbiology, Faculty of Medicine, Kuwait University 37603, Safat, Kuwait; 6 Mycology Reference Laboratory, Spanish National Centre for Microbiology, Madrid, Spain; 7 Centro de Investigación Biomédica en Red de Enfermedades Infecciosas, Instituto de Salud Carlos III 38176, Madrid, Spain; 8 Department of Microbiology and Infection Control, Statens Serum Institut 4326, Copenhagen, Denmark; 9 Department of Clinical Microbiology, Copenhagen University Hospital, Copenhagen, Denmark; 10 National Mycology Reference Laboratory, Public Health England 373127 , Bristol, United Kingdom; 11 Centre for Infectious Diseases and Microbiology Laboratory Services, Westmead, Australia; 12 Medical Mycology Unit, Department of Microbiology, Vallabhbhai Patel Chest Institute, University of Delhi 72916 , Delhi, India; 13 National Reference Laboratory for Antimicrobial Resistance in Fungal Pathogens, Vallabhbhai Patel Chest Institute, University of Delhi 28742, Delhi, India; 14 Division of Infectious Diseases, Mount Auburn Hospital, Cambridge, MA & Harvard Medical School 1811 , Boston, Massachusetts, USA; 15 University of Cologne, Faculty of Medicine, Institute of Translational Research 14309, Cologne, Germany; 16 Department I of Internal Medicine, Excellence Center for Medical Mycology, University of Cologne 14309, Cologne, Germany; 17 Laboratoire de Santé Publique du Québec, Institut National de Santé Publique du Québec, Sainte-Anne-de-Bellevue, Canada; 18 Department of Pathology, University of Texas Southwestern Medical Center 12334, Dallas, Texas, USA; 19 Department of Mycology, Centre Hospitalier Universitaire de Rennes, Centre National de Référence Aspergilloses chroniques, ECMM Excellence Center in Mycology 36684, Rennes, France; 20 Unitat de Micologia i Microbiologia Ambiental, Facultat de Medicina i Ciènces de la Salut, Universitat Rovira i Virgili 674469 , Reus, Spain; 21 Infectious Disease Research Program, Department of Pediatric Hematology and Oncology, University Children‘s Hospital Muenster, Münster, Germany; 22 Oniris, VetAgroBio Nantes, IRF, SFR ICAT, Université d'Angers 26995, Angers, France,; 23 Laboratory Diagnostic Center, RWTH Aachen University Hospital, Aachen, Germany; 24 Centre for Infectious Diseases and Microbiology Laboratory Services, Westmead, Australia; 25 Royal Botanic Gardens, Kew 41949, Richmond, Surrey, United Kingdom; 26 Natural History Museum 4946, London, United Kingdom; 27 University of Southampton 7423, Southampton, United Kingdom; 28 Jilin Agricultural University 85112, Changchun, China; 29 St John’s Institute of Dermatology, King’s College London 388741 , London, United Kingdom,; 30 Division of Infectious Diseases, Medical University of Graz, Graz, Austria; 31 Translational Medical Mycology Research Unit, Medical University of Graz 31475, Graz, Austria; 32 Department of Botany, Charles University, Prague, Czechia; 33 Department of Medical Microbiology, Faculty of Biology, University of Warsaw, Warsaw, Poland; 34 Institute of Environmental Science (CML), Leiden University 4496, Leiden, the Netherlands; 35 National Mycology Reference Centre, SA Pathology 10107, Adelaide, Australia; 36 School of Biological Sciences, Faculty of Sciences Engineering and Technology, University of Adelaide 1066, Adelaide, Australia; 37 Public Health Ontario 153300, Toronto, Ontario, Canada; 38 Department of Laboratory Medicine and Pathobiology, University of Toronto 7938, Toronto, Canada; 39 National Institute of Allergy and Infectious Diseases, National Institutes of Health 2511, Bethesda, Maryland, USA; 40 Centers for Disease Control and Prevention 1242, Atlanta, Georgia, USA; 41 Microbiology and Molecular Genetics, Biomedical Laboratory Diagnostics, Michigan State University 3078, East Lansing, Michigan, USA; 42 Westerdijk Fungal Biodiversity Institute of the KNAW, Utrecht, the Netherlands; 43 University of Pittsburgh Medical Center 6595, Pittsburgh, Pennsylvania, USA,; 44 Department of Dermatology, Peking University First Hospital, Peking University 26447, Beijing, China; 45 Sydney Infectious Diseases Institute, Faculty of Medicine and Health, University of Sydney, Sydney, Australia; 46 Bioinformatics, Helmholtz Institute for One Health 657809 , Greifswald, Germany; 47 CONICET (Consejo Nacional de Investigaciones Científicas y Tecnológicas), Hospital JM Ramos Mejía 62873 , Buenos Aires, Argentina; 48 Division of Clinical Microbiology, Mayo Clinic 6915, Rochester, Minnesota, USA; 49 Public Health Wales Microbiology, Cardiff, United Kingdom; 50 McGovern Medical School, Division of Infectious Diseases, University of Texas Health Science Center 12340, Houston, Texas, USA; 51 Center for Innovative Therapeutics and Diagnostics 630443, Richmond, Virginia, USA; 52 University of Maryland School of Medicine 12264, Baltimore, Maryland, USA; Vanderbilt University Medical Center, Nashville, Tennessee, USA

**Keywords:** nomenclature, * Candida auris*, candidiasis, fungal disease

## REPLY

We thank the authors for their interest in our paper advocating for the standardization of naming fungal pathogens and diseases. We acknowledge their proposal to rename the infection caused by *Candida auris*, following the proposal to rename the causal agent as *Candidozyma auris*, from “candidiasis” to “candidozosis” or “candidozymiasis,” based on the taxonomic and biological arguments presented in their letter.

While we value this intriguing proposal, we believe that renaming *Candida auris* to *Candidozyma auris*, along with changing the associated disease name beyond candidiasis, is premature and not in the interests of users. Current fungal classifications are largely driven by DNA sequencing and phylogenetic analysis, but the materials selected for analysis and the methodologies used can vary considerably, raising questions about the reproducibility of taxonomic findings. The proposed reclassification of *Candida auris* to *Candidozyma auris* relies on a single study that examined only one strain of *Candida auris* (1); thus, the taxonomic finding in the paper lacks robustness due to insufficient sampling, a lack of phylogenetic analyses validated by other investigators, and unclear ecological distinctions. The authors of the study state that the name change is “officially reported”, but there is no regulating body for fungal taxonomy; this decision represents a conclusion by the authors of the paper and has not been debated by, for example, a Working Group (WG) of the International Committee on the Taxonomy of Fungi (ICTF). The remote position of *Candida auris* compared to *Candida albicans* has been confirmed in some phylogenomic studies (2, 3). These two species are positioned within the same order but as members placed in different families (*Metschnikowiaceae* vs. *Debaryomycetaceae*, respectively). *Candidozyma* is as yet unstable, as more species are being added (1), and the generic size compared to, e.g. *Clavispora* remains to be discussed, as current taxonomic distances between genera of *Metschnikowiaceae* are very small, and so these might also be treated as subgenera or sections rather than separate genera. Moreover, given the significant genetic diversity observed across the various clades of *Candida auris* (currently six clades proposed) (4, 5), as well as different antifungal susceptibility profiles (6), there are concerns that these distinct clades may eventually be reclassified as separate species, resulting in yet more unnecessary and interminable name changes that could destabilize the species classifications. In light of the clinical importance and widespread recognition of *Candida auris* in medical and public health literature, any proposed renaming should be supported by further robust and reproducible genomic and phylogenetic evidence leading to broad taxonomic agreement. For these reasons, we advocate maintaining the name *Candida auris* until consensus is reached by an international WG.

We further argue that retaining the term “candidiasis” for infections caused by *Candida auris* is essential to ensure clear communication and understanding within the scientific and medical communities. Although *Candida auris* does exhibit several distinctive biological traits compared to other *Candida* species, these distinctions do not warrant a name change or any associated disease name. In fact, characteristics, such as high transmissibility, the ability to persist in hospital environments, the capacity to cause healthcare-associated outbreaks, and emerging drug resistance, are shared with several other *Candida* species, including *Candida parapsilosis* (7). *Candida auris* behaves similarly to other *Candida* species. It is an opportunistic fungal pathogen that exists in the environment and can colonize humans, clinically causing candidemia, urinary tract infections, and deeply invasive diseases (8). The diseases it causes align closely with the clinical features of candidiasis and can be appropriately treated according to recently established global guidelines for candidiasis (9). An analogy with the *Mucorales*, where phylogenetically remote agents cause similar disease patterns collectively referred to as mucormycosis, is instructive. Furthermore, more than 30 *Candida* species causing candidiasis have been proposed for renaming, either by adopting sexually typified names or phylogenetic names such as *Candidozyma*. It is impractical to rename diseases in response to every new designation, especially when the associated infections exhibit characteristics and disease spectra that are consistent with candidiasis. It has to be remembered that the purpose of names is primarily to facilitate unambiguous communication between users of names and that the introduction of new generic names, in particular, is often not in users’ interests. There are many cases where genera include very large numbers of species or subspecies falling into different clades and are retained in that genus in the best interests of users of names by using infrageneric categories (10).

*Candida auris* is listed as a critical priority fungal pathogen by the World Health Organization (11). Its emergence in 2009 further underscores the need for continued vigilance in managing candidiasis, and *Candida auris* has now become a significant cause of candidiasis in healthcare settings worldwide. Prematurely renaming it to *Candidozyma auris* and altering the associated disease designation beyond candidiasis will undermine the established epidemiological understanding and tracking, regulations, clinical diagnosis, treatment, infection control, and research efforts on this critical fungal pathogen.

Finally, while a proposed name change may be logically justified from a taxonomic standpoint if narrow genetic and species concepts are adopted, implementing such changes in clinical settings must be approached with caution and thorough consideration. To address the evolving field of fungal taxonomy and nomenclature with clinical name changes, the ISHAM WG on the nomenclature of clinical Fungi, a multidisciplinary team composed of clinicians, public health professionals, clinical microbiologists/medical mycologists, and taxonomists, is currently developing practical criteria to guide the implementation of name changes of medically important fungi for clinical use (12). The WG applies a structured, evidence-based approach that incorporates taxonomic, epidemiological, clinical, laboratory, public health, and regulatory perspectives to facilitate implementation of scientifically justified fungal name changes into clinical practice, while safeguarding patient care and advancing our understanding of fungal taxonomy. Our WG is open to anyone interested in fungal nomenclature, and participation is welcome via the ISHAM website (https://www.isham.org/working-groups/fungal-nomenclature/).
